# HIV Capsid is a Tractable Target for Small Molecule Therapeutic Intervention

**DOI:** 10.1371/journal.ppat.1001220

**Published:** 2010-12-09

**Authors:** Wade S. Blair, Chris Pickford, Stephen L. Irving, David G. Brown, Marie Anderson, Richard Bazin, Joan Cao, Giuseppe Ciaramella, Jason Isaacson, Lynn Jackson, Rachael Hunt, Anne Kjerrstrom, James A. Nieman, Amy K. Patick, Manos Perros, Andrew D. Scott, Kevin Whitby, Hua Wu, Scott L. Butler

**Affiliations:** 1 Pfizer Global Research and Development, La Jolla Laboratories, San Diego, California, United States of America; 2 Antiviral Biology, Pfizer Global Research and Development, Sandwich, Kent, United Kingdom; 3 Structural Biology Department, Pfizer Global Research and Development, Sandwich, Kent, United Kingdom; University of Geneva, Switzerland

## Abstract

Despite a high current standard of care in antiretroviral therapy for HIV, multidrug-resistant strains continue to emerge, underscoring the need for additional novel mechanism inhibitors that will offer expanded therapeutic options in the clinic. We report a new class of small molecule antiretroviral compounds that directly target HIV-1 capsid (CA) via a novel mechanism of action. The compounds exhibit potent antiviral activity against HIV-1 laboratory strains, clinical isolates, and HIV-2, and inhibit both early and late events in the viral replication cycle. We present mechanistic studies indicating that these early and late activities result from the compound affecting viral uncoating and assembly, respectively. We show that amino acid substitutions in the N-terminal domain of HIV-1 CA are sufficient to confer resistance to this class of compounds, identifying CA as the target in infected cells. A high-resolution co-crystal structure of the compound bound to HIV-1 CA reveals a novel binding pocket in the N-terminal domain of the protein. Our data demonstrate that broad-spectrum antiviral activity can be achieved by targeting this new binding site and reveal HIV CA as a tractable drug target for HIV therapy.

## Author Summary

Although the current standard of care for Human Immunodeficiency Virus (HIV) is high, viral resistance has emerged to every drug currently in the clinic, in some cases rendering the entire class ineffective for patients. A new class of antiretroviral drugs would be effective against strains of HIV-1 that are resistant to any existing drug and would expand the therapeutic options available to patients. Capsid is the primary structural protein of HIV and a critical part of the viral replication cycle, both in the assembly of viral particles and in the infection of host cells. We report a new class of antiretrovirals that targets HIV-1 capsid and demonstrate that it is active at two critical stages in the viral replication cycle. These compounds were consistently effective against a range of clinical strains of HIV-1, from various sub-types, as well as HIV-2. Finally, the compounds bind in a unique pocket on capsid that has not previously been highlighted as a drug binding site. We believe this new class of antiretrovirals can serve as a starting point for the development of a new generation of HIV-1 therapeutics and, more generally, underscores the potential of capsid as a therapeutic target.

## Introduction

Highly active antiretroviral therapies (HAART) against human immunodeficiency virus type 1 (HIV-1) have proven in recent years to be extremely effective at reducing viral load and significantly delaying disease progression [1]. However, there remains a pressing need to discover and develop new classes of HIV inhibitors. The virus continues to acquire resistance to currently administered antiretroviral drugs and the rate of transmitted resistance is increasing [2], [3]. The discovery of compounds that inhibit the replication of HIV-1 via new mechanisms offers the best hope of generating drugs that are active against all HIV-1 variants in the clinic. The potency of these compounds would not be affected by mutations that confer resistance to existing therapies [4].

The capsid protein (CA) of HIV-1 plays critical roles in both late and early stages of the viral replication cycle and is widely viewed as an important unexploited therapeutic target [4], [5], [6]. At the earliest stages of particle assembly, the interactions between CA domains of the Gag polyprotein help drive the formation of immature particles at the membrane of host cells [7]. After the release of immature particles from infected cells, proteolytic processing of the Gag polyprotein is completed, leading to capsid assembly and formation of the mature virus. During assembly, the viral RNA genome is packaged into a capsid particle composed of a lattice of CA protein hexamers that form a distinct fullerene cone shaped particle [8]. After virus fusion with a target cell, the core is released into the cytoplasm and CA is thought to undergo a controlled disassembly reaction in order for reverse transcription of the viral genome to occur properly [9].

The HIV-1 CA protein has attracted increased interest as a drug discovery target in recent years. A small molecule, CAP-1, and two versions of a peptide inhibitor, CAI and NYAD-1, have been described that target HIV-1 CA in vitro and appear to interfere with CA function in infected cells [10], [11], [12]. In addition, high resolution structural data on the hexameric lattice that forms the full core structure has been reported [13], [14]. These structures illustrate the distinct roles and importance of inter-subunit interfaces in the CA complex and have shed some light on the potential mechanisms of previously reported CA assembly inhibitors.

Here we describe a novel series of antiviral compounds that target HIV-1 CA in infected cells and appear to interfere with both the viral uncoating process and the formation of infectious particles. Mechanism-of-action and *in vitro* resistance studies of this series are described. A high resolution co-crystal structure has been determined and illustrates a novel binding pocket in the N-terminal domain (NTD) of HIV-1 CA that is distinct from any previously described. We demonstrate that targeting this new binding pocket with small molecules results in broad-spectrum antiviral activity. This study provides the starting point, where structure-based drug design is a viable option, for the development of a new class of HIV therapeutics.

## Results

### *In vitro* Antiviral Activity

**Figure 1:**
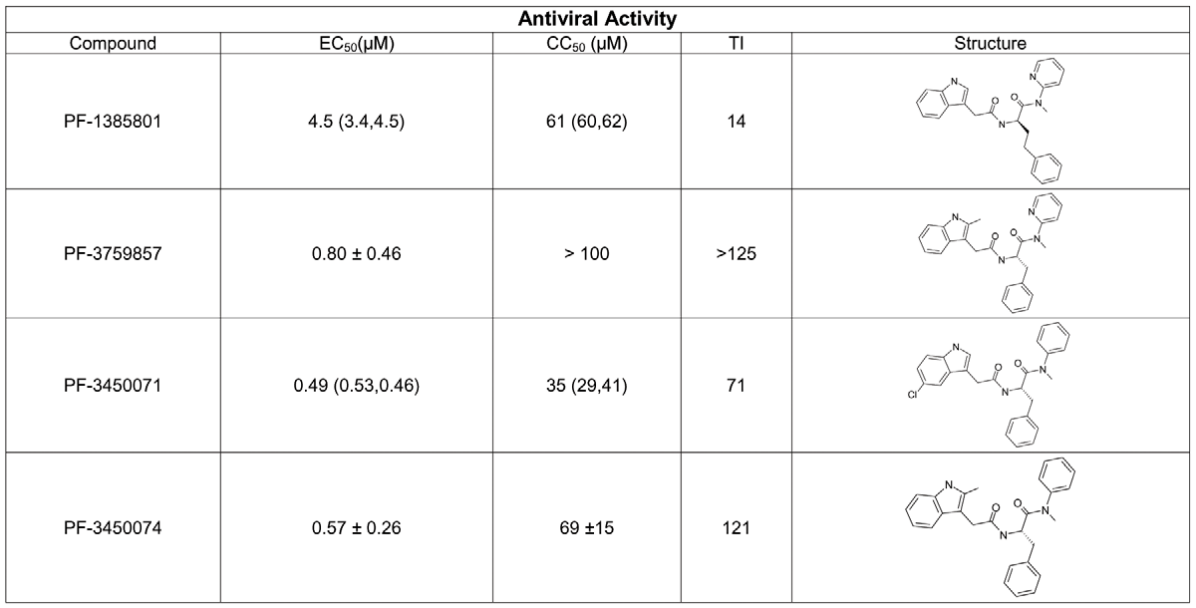
Structures and antiviral activities of inhibitors that target HIV-1 capsid. Antiviral activity was determined in CPE assays after infection of MT-2 cells with HIV-1 NL4-3. Results represent the mean ± standard deviations from 4–6 experiments, or the mean with individual values from 2 experiments. TI, therapeutic index is the ratio of mean CC_50_: mean EC_50_.

PF-1385801 was identified as a hit in a high throughput screen for inhibitors of HIV replication [15]. Several analogs of this compound demonstrated activity in antiviral assays using the MT-2 T-cell line and HIV-1 NL4-3. PF-1385801 inhibited HIV-1 replication with a 50% effective concentration (EC_50_) of 4.5 µM and exhibited a 50% cytotoxic concentration (CC_50_) of 61 µM, resulting in a therapeutic index (TI, CC_50_/EC_50_) of 14 (Fig. 1). More potent analogs were subsequently designed and synthesized, as detailed in Fig. 1.

**Figure 2:**
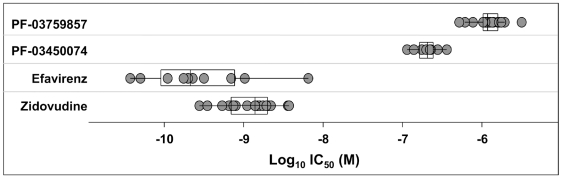
Activity of inhibitors against diverse strains of HIV-1. The spectrum of antiviral potencies was determined for two capsid inhibitors, efavirenz, and zidovudine against a panel of HIV isolates including clinical isolates and laboratory adapted strains. Each dot in the figure represents the geometric mean EC_50_ value for a single HIV isolate. The variability of the antiviral spectrum is further illustrated for each compound with a superimposed “box whisker” plot. The box represents the inter-quartile range and the whiskers highlight the 95% confidence interval. The vertical line in the box represents the geometric mean of the EC_50_ values.

A key issue in the development of novel HIV drugs is the potential therapeutic spectrum (consistency of activity across multiple strains of HIV). To address this, two of the compounds were evaluated in antiviral assays using peripheral blood mononuclear cells (PBMCs) infected with a diverse set of HIV-1 lab strains and clinical isolates covering 6 clades and both X4 and R5 tropic viruses (Fig. 2). PF-3450074 and PF-3759857 were active against all strains of HIV-1 tested with median EC_50_ values of 0.207 (range 0.113 to 0.362 µM) and 1.17 (range 0.51 to 3.17) µM, respectively (Fig. 2 and Tables S1 and S2). In addition, PF-3759857 was active against HIV-2 with an EC_50_ of 4.7 µM. This tight spectrum of activities against HIV-1 compared well with the marketed drugs, AZT, a nucleoside analog, and efavirenz (EFV), a non-nucleoside reverse transcriptase inhibitor (NNRTI) (Fig. 2 and Tables S3 and S4).

### *In vitro* Resistance Studies to Identify the Antiviral Target

To positively identify the antiviral target of this class of inhibitors, PF-1385801 resistant viral variants were selected in *in vitro* serial passage experiments. Sequence analysis of cDNAs derived from resistant viral variants selected in the presence of PF-1385801 revealed a single mutation, T107N, located in the NTD of the CA protein. No mutations associated with resistance selection were identified in the integrase or reverse transcriptase coding sequences. Recombinant HIV-1 NL4-3 virus encoding the T107N substitution in HIV-1 CA exhibited an 11-fold reduction in susceptibility to PF-1385801 when compared to wild-type NL4-3 (Table 1). Similar reductions in susceptibility were observed for PF-3450071 (6-fold) and PF-3759857 (10-fold), while no shift was observed for EFV (NNRTI) or the integrase inhibitor AG-110079. In subsequent *in vitro* resistant virus selection experiments using a compound with a larger TI, PF-3759857, additional substitutions were identified in CA (T107N, H87P, Q67H, K70R and L111I), after 53 days in culture. Virus isolated from the final passage of this experiment showed a >60-fold reduction in susceptibility to PF-3759857 and recombinant NL4-3 virus encoding the five substitutions (5M) exhibited a >40-fold reduction in susceptibility to PF-3450074 (up to the cytotoxicity limit of the compound). Wild-type NL4-3, the T107N mutant, and the 5M mutant virus all exhibited similar levels of infectious virus production from transfected cells and comparable levels of viral replication in reporter gene-based infection assays (in the absence of compound), suggesting that the substitutions do not significantly impair replication capacity. However, more detailed studies, such as direct competition assays are required to accurately determine if the substitutions affect replication fitness.

**Table 1:** *In vitro* anti-viral activity against HIV-1 capsid wt and T107N mutant.

	EC_50_ (µM) against HIV NL4-3^a^			
Compound	Wild type	T107N	FC^b^	P^c^
PF-1385801	2.7±1.4	28±7.1	11	0.015
PF-3450071	0.72±0.23	4.5±1.1	6	0.014
PF-3759857	2.5±1.1	24±9.5	10	0.014
EFV	0.0012±0.00013	0.00064±0.00002	1	nd
AG-110079	0.0037±0.0016	0.0038±0.0031	1	nd

a EC_50_ values were determined in full replication assays (not CPE).

b FC = Fold-change calculated by dividing the mutant (T107N) EC_50_ by the WT EC_50_.

c P = P-value, determined using the student's T-test.

These results demonstrate that mutation(s) in HIV-1 CA (e.g. T107N) are sufficient to confer resistance to this new class of inhibitors and demonstrate that HIV-1 CA is the target in infected cells. Although additional work is needed to determine the individual contributions of the mutations identified in addition to T107N, it appears that more than one mutation is required to generate high levels of resistance to this inhibitor class. While it is a definitive tool for target identification, it should be noted that *in vitro* selection can be used to generate resistant mutants to any known antiviral and does not accurately predict the clinical barrier to resistance, which is a function of several in vivo parameters, including the pharmacokinetics of the compound.

### Inhibition of Early Events in the HIV-1 Replication Cycle

To determine the stage of the replication cycle targeted by this new class of compounds, PF-3450071 and PF-3450074 were evaluated in single cycle infection assays. Such assays monitor the early steps of infection up to the integration of the viral cDNA into the host cell chromosome and expression of that sequence. PF-3450071 and PF-3450074 were tested in parallel infections with single-cycle HIV-1 virus packaged with either wild type HIV-1 envelope (NL4-3 pseudovirus) or vesicular stomatitis virus glycoprotein (VSVG), an envelope that allows viral entry by an alternative mechanism (VSV pseudovirus). In addition, three compounds with known mechanisms, AMD3100 (HIV CXCR4/entry inhibitor), EFV (NNRTI/early mechanism) and NFV (protease inhibitor (PI)/late mechanism) were evaluated in the assays. PF-3450071 and PF-3450074 were active against both the NL4-3 and VSV pseudoviruses (Table 2). This inhibition profile was similar to EFV. The profile was distinct from that of AMD3100, which inhibited the HIV-1 envelope NL4-3 pseudovirus, but not the VSVG pseudovirus, and NFV which was not active in the assay (Table 2). These data indicate that PF-3450071 and PF-3450074 act early in the HIV-1 replication cycle at a step following HIV-1 envelope-mediated entry.

**Table 2:** Activity of antiviral compounds in selected assays.

Compound (Target)	Activity in specified assay (EC_50_ µM)^a^			Stage of action
	NL43 Pseudovirus	VSV-G Pseudovirus	Production/Infection	
PF-3450071 (capsid)	0.51	0.39	0.78	Early/Late
PF-3450074 (capsid)	0.55	0.32	0.33	Early/Late
AMD3100 (viral entry)	0.002	>100	n.d.	Entry
Efavirenz (RT)	0.001	0.001	>1	Early
NFV (Protease)	>10	n.d.	0.077	Late

a Antiviral activity was determined after infection of HeLa CD4 LTR/beta-Gal cells with HIV single-cycle infectious viruses packaged with either the NL4-3 or VSV-G envelope proteins.

### Elucidating the Early Stage Mechanism

**Figure 3:**
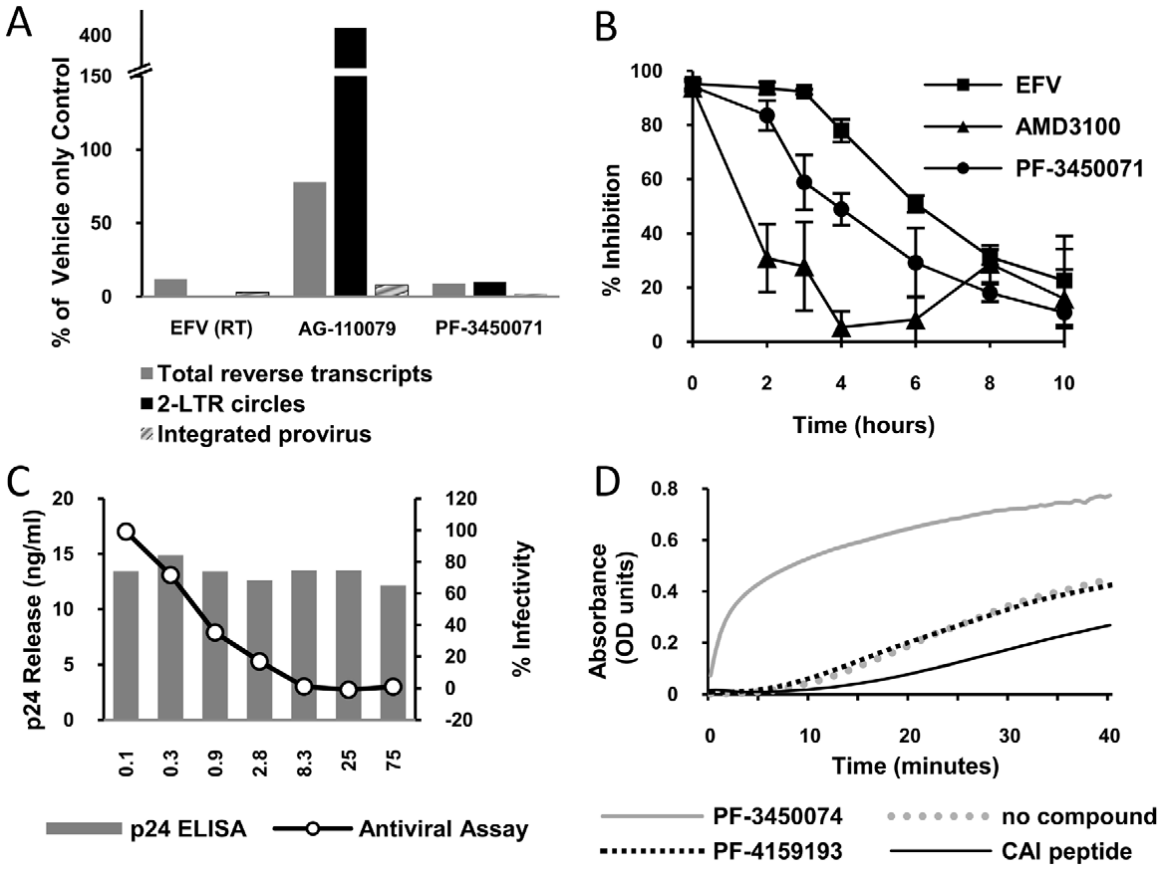
Identification of mechanism of action. a) HIV DNA quantification. A Taqman quantitative PCR assay was performed to determine the effect of test compounds on the levels of total HIV DNA, 2-LTR circles and integrated provirus generated during infection. The data is presented as a percentage of amounts observed in an untreated control tested in parallel. b) Time-of-addition experiment. HeLa CD4 LTR/beta-Gal reporter cells were infected with HIV-1 (NL43) and test compounds (at 2×EC _90_) were added at different times after infection. Reporter signal was monitored at 72 h post-infection as a measure of replication. c) Effect of PF-3450074 in a virus production assay. 293T cells transfected with pNL43 were incubated in varying concentrations of PF-3450074. After 48 hours supernatants were transferred to HeLa CD4 LTR/beta-Gal reporter cells. The line graph shows the percentage of β galactosidase production compared with a mock treated control. Solid bars indicate the concentration of HIV capsid protein (p24) secreted by the 293T cells at each concentration tested. d) Kinetics of CA multimerization. HIV CA spontaneously multimerizes in the presence of high salt, causing a change in optical density. Test compounds were incubated with CA protein and multimerization was initiated with the addition of concentrated salt solution.

To begin dissecting the early events of infection, DNA was isolated from single cycle infections of MT-2 cells in the presence or absence of inhibitors and analyzed by quantitative PCR. Primer sets were used that detect total viral cDNA produced, 2-LTR circles (a nuclear episomal form of the viral cDNA), or provirus (the viral genome integrated into host cell chromosomes) as described previously [16]. PF-3450074 inhibited the accumulation of total viral cDNAs (9% of control) and as result inhibited the levels of integrated provirus DNA measured (2% of control) (Fig. 3a). This profile was similar to that observed for the RT inhibitor EFV, indicating that PF-3450074 either inhibits reverse transcription or a step prior to it. In contrast, the integrase inhibitor, AG-110079, resulted in a 4-fold increase in 2-LTR circle accumulation and minimal reduction in total viral cDNA (78% of control) (Fig. 3a). As the profile for PF-3450074 did not match that of the integrase inhibitor control, integrase inhibition can be ruled out as a possible mechanism.

To further analyze the mechanism of action, the inhibition profile of PF-3450071 was compared to that of AMD3100 and EFV in synchronized time-of-addition experiments, where inhibitors are added a various time points following infection to monitor when the susceptible step has occurred. The viral entry inhibitor, AMD3100, showed a dramatic early loss in activity, even within the first two hours of infection. The NNRTI, EFV, retained the majority of inhibitory activity (>78%) when added up to 4 hours after infection and lost activity thereafter, with a mid-point around 6 hours after infection. PF-3450071 displayed a profile that was distinct from both comparators. The compound maintained the majority of its inhibitory activity (>84%) when added up to 2 hours after infection, and lost significant levels of activity when added 3 or more hours after infection (Fig. 3b). These results suggest that this new class of HIV-1 inhibitor targets an early step in the virus replication cycle, after viral entry but before reverse transcription, possibly viral uncoating. Consistent with these data, PF-3450074 did not inhibit recombinant HIV-1 RT in standard biochemical assays (IC_50_>100 µM).

### Inhibition of Late Events in the HIV-1 Replication Cycle

Virus production-infectivity assays were conducted to determine if compounds affect the late stages of viral replication. Infectious virus was expressed from transfected producer cells in the presence or absence of compound and the infectivity of the resulting virus was tested by diluting the supernatant on to an indicator cell line. As infectious virus is produced from a transfected DNA, bypassing the early stages of the replication cycle, only inhibitors targeting the later stages of viral replication (i.e. post-integration) should show activity in the assay. We tested two compounds (PF-3450071 and PF-3450074) in the viral production-infectivity assay using NFV (PI) and EFV (NNRTI) as late stage and early stage inhibitor controls. Both PF-3450071 and PF-3450074 inhibited the production of infectious virus with EC_50_ values of 0.78 and 0.33 µM, respectively (Table 2). As expected the HIV-1 PI, NFV, inhibited infectious virus production while the RT inhibitor, EFV was not active in the assay (Table 2). Notably, the overall levels of Gag proteins released into the supernatant of transfected cells were not affected by PF-3450074, as measured by p24 ELISA (Fig. 3c). Thus, this series does not inhibit HIV-1 particle production but renders the nascent particles noninfectious.

Western blot analysis of viral supernatants with antibodies directed against the HIV-1 CA protein (p24) showed that PF-3450071 did not affect HIV-1 Gag proteolytic processing (Fig. S1), which demonstrates that the compounds do not inhibit HIV-1 protease or virion maturation in the manner described for inhibitors such as Bevirimat (PA-457) [17] and PF-46396 [18]. Collectively, these data suggest an effect of the series on proper assembly of fully processed CA protein in nascent viral particles.

### Disruption of the Formation of Native-Like Particles

**Figure 4:**
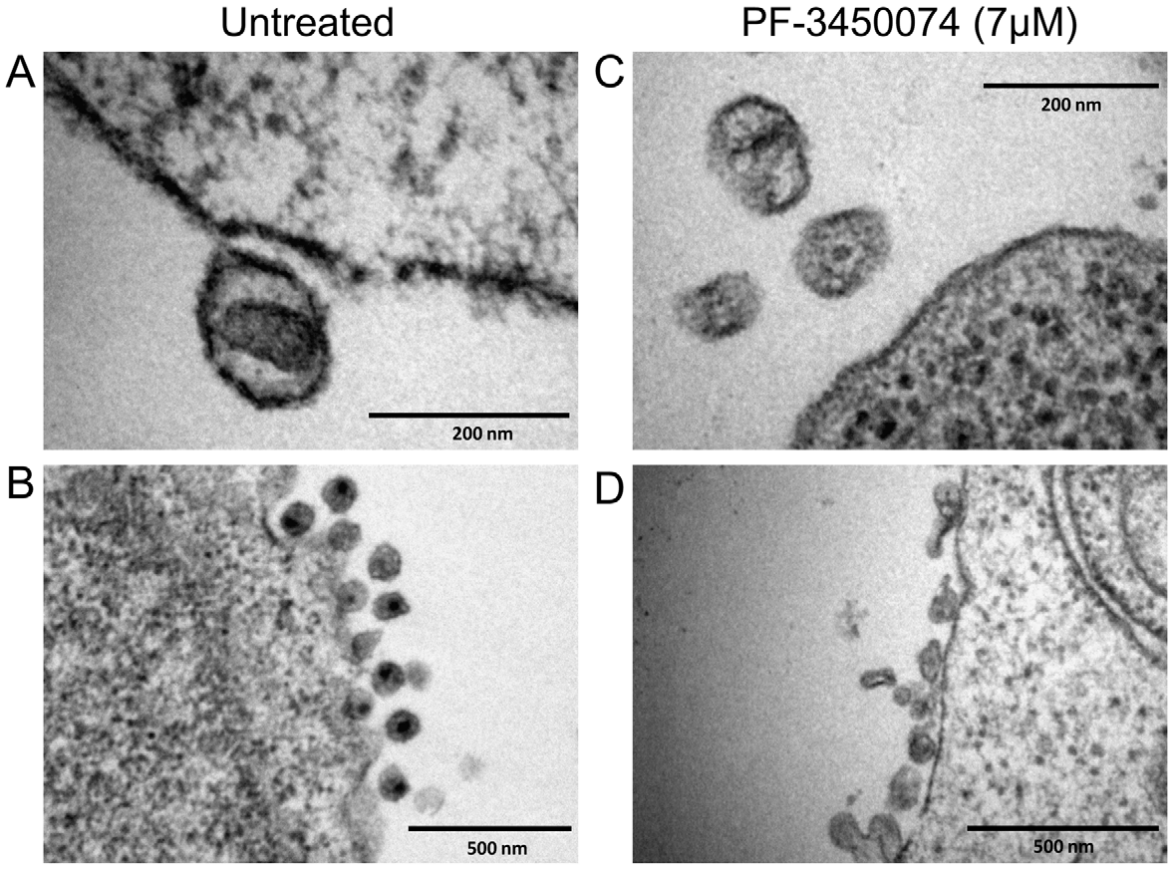
EM analysis of the effects of PF-3450074 on nascent viral particles. PBMCs were infected with HIV NL4-3 in medium containing either no compound or 7µM PF-3450074. The cells and resulting viral products were processed and observed by transition electron microscopy. Close in view of an untreated native-like particle (a) reveals the central conical capsid structure, while a wider view (b) shows the high level of uniformity in morphology and size among untreated particles. Close in view of PF-3450074-treated particles (c) shows consistent loss of the central capsid density, and a wider view (d) illustrates a much wider range in particle morphology and size.

To observe the effects of this class of compound on the morphology of nascent viral particles, PBMCs were infected with HIV NL4-3 either in the absence or presence of PF-3450074 at 7 µM (∼10× EC_50_). The cells and resulting viral products were fixed and treated for transmission electron microscopy (TEM). In untreated infections, approximately 66% of particles (78/118 observed) were native-like, as defined by being ∼100nm in diameter and having a distinct central density representative of a mature capsid core). A close in view of a native-like particle from an untreated culture (Fig. 4a) clearly illustrates the conical capsid core in the center of the virion. A wider view shows the high level of uniformity among the particles produced in the untreated infections (Fig. 4b). Both native-like and apparent immature particles are present at consistent shape and size. In contrast, native-like particles were not observed in PF-3450074-treated infections. The particles produced in PF-3450074-treated infections lack a clear central density representative of a mature capsid (Fig. 4c). Consistent with the observation that these compounds do not affect p24 levels, the number of particles in PF-3450074-treated infections appears similar to untreated, however the morphology and size of particles is highly variable (Fig. 4d).

### Compound Effects on CA Multimerization

The simplest explanation to reconcile the data above involves a model where this new class of inhibitors targets both early (viral core uncoating) and late (viral core assembly) events in the replication cycle by affecting CA – CA interactions and thus core stability. To study the effects on capsid assembly, we evaluated the inhibitors in *in vitro* CA multimerization assays [10]. Such assays can be used to measure the effect of compounds on the rate of formation of higher order CA multimers or tubes that are widely thought to represent many aspects of native core structure. Addition of PF-3450074 results in a significant increase in the rate of CA multimerization (Fig. 3d). In contrast, a structural analogue with no antiviral activity, PF-4159193 (Fig. S2), did not affect the kinetics of CA multimerization, indicating that this profound effect is correlated with antiviral activity and not a general physical effect of this series. As we have reproduced with the CAI peptide (Fig. 3d), all of the previously reported HIV CA assembly inhibitors decreased the rate of multimerization in this assay [10], [11], [12]. These data demonstrate indirectly that PF-3450074 interacts with HIV-1 CA and further suggest a mechanism that is fundamentally distinct from previously reported HIV CA inhibitors.

### High Resolution Co-Crystal Structure Reveals a Novel Binding Pocket

**Figure 5:**
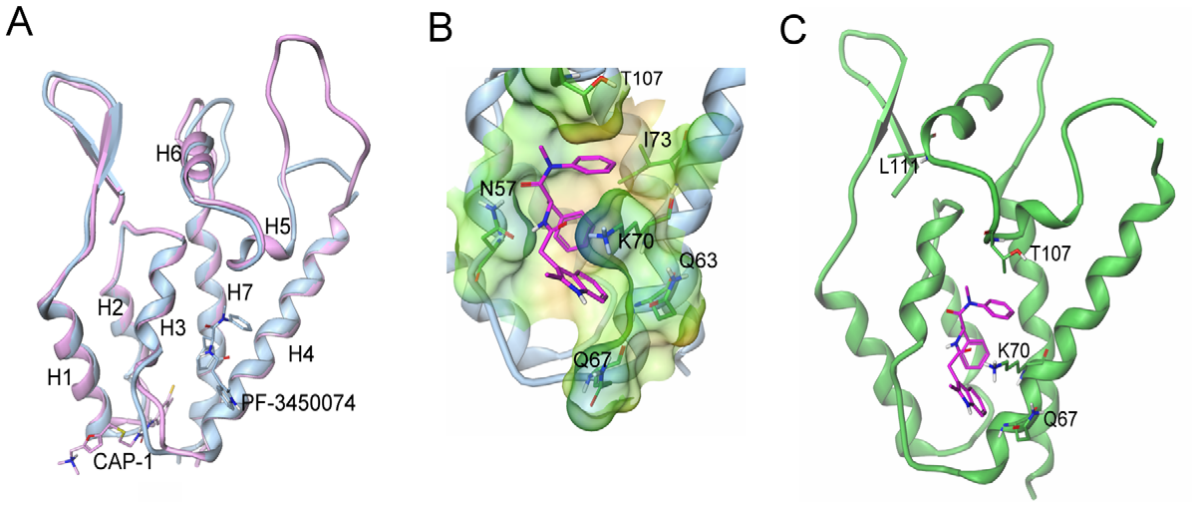
Structure of the novel inhibitor binding site and context in the NTD. a) Overlays of capsid structures with PF-3450074 in blue and CAP-1 in pink bound to capsid N-terminal domain; b) Close up view of PF-3450074 site (binding site residues labelled in black, R1-3 sub-pockets labelled in purple). In the R1 sub-pocket PF-3450074 makes hydrophobic interactions with Ile-73, Ala-105, Thr-107, Tyr-130 and a stacking interaction with the side chain of Asn-53. The benzyl group R2 makes a number of hydrophobic interactions in a sub-pocket formed by the side chains of Met66, Leu69, Val59, Ile-73 and Leu-56. The R3 sub-pocket is only partially occupied by the indole group, forming interactions with the side chains of Met-66, Gln-67, Lys-70 and Gln-63 amide. The indole NH forms a hydrogen bond interaction with the side chain amide of Gln-67 via a water molecule, while an Asn-57 forms a key hydrogen bond with the cis amide bond of PF-3450074. c) Location of resistant mutations (purple) in relation to PF-3450074 capsid binding site. (PDB ID code 2XDE).

To further understand the mode of action of this novel class of compounds, we determined the crystal structure of HIV-1 CA NTD protein in complex with PF-3450074 using a CA protein construct that contained a single glycine residue in place of the cyclophilin binding loop (residues 87–99). Although the cyclophilin binding loop is important for viral infection, it is not required for proper HIV CA protein folding and in vitro multimerization function [8]. Binding affinity of PF-3450074 to the crystallographic construct (K_d_ = 3.42 µM) is similar to that observed for both full length wild type CA (K_d_ = 2.79 µM) and isolated wild type NTD (K_d_ = 2.24 µM), as measured by isothermal titration calorimetry. Using this construct, the co-crystal structure of PF-3450074 was solved with the NTD of HIV-1 CA to 1.8 Å resolution. The structure of the complex showed that the overall fold of the CA protein is the same as previously described CA structures [19], [20], [21] and illustrates that neither the compound, PF-3450074, nor the loop deletion caused any significant shifts in the protein structure (Fig. 5a).

PF-3450074 occupies a preformed pocket in the HIV-1 CA NTD bounded by helices 3, 4, 5 and 7 (Fig. 5a). The R1 and R2 aromatic moieties of the compound occupy two hydrophobic sub-pockets and provide most of the key interactions which anchor the compound to the NTD (Fig. 5b). The indole substituent protrudes from the NTD R3 sub-pocket close to Lys70. The binding site for PF-3450074 is distinct from the sites targeted by CAP-1 and CAI/NYAD-1 [10], [11], [22], [23], [24]. These results confirm that PF-3450074 directly binds HIV CA and are consistent with this class of inhibitors acting via a unique mechanism. Coordinates are stored in the Protein Data Bank (PDB ID code 2XDE).

## Discussion

We describe a novel class of inhibitors that target HIV CA by a unique mechanism that interferes with both early and late events in the viral replication cycle. HIV CA plays an essential role in several stages of viral replication and is viewed as an important, yet unexploited target for therapeutic intervention [4], [5], [6]. This new series demonstrates that small molecules targeting HIV CA can have potent broad-spectrum antiviral activity. We demonstrate directly that HIV CA is the antiviral target of these inhibitors in infected cells by showing that mutations in HIV CA confer resistance to several members of the series. EM analysis shows that the series profoundly affects the morphology of nascent HIV particles. We demonstrate that the compounds affect CA protein multimerization *in vitro* and we have elucidated details of the novel compound binding site on HIV CA by solving a co-crystal structure of a compound from the series bound to the NTD of the protein. Our data strongly suggest that this new series of inhibitors targets HIV CA function during both the virion uncoating and viral core assembly processes.

Previous studies have described two molecules that target HIV-1 CA assembly in vitro, CAP-1 (a small molecule) and CAI (a dodecapeptide) [10], [11], [12]. CAP-1 acts in the late stage of viral replication and does not inhibit HIV-1 infection when added to pre-formed HIV-1 particles. A cell-permeable derivative of CAI, NYAD-1, inhibits formation of both immature and mature HIV-1 virus particles as well as early events in the replication cycle at low micromolar concentrations. The properties of the new class of CA inhibitors described in this study are clearly distinguished from those of other HIV-1 inhibitors, including previously described CA inhibitors. Unlike CAP-1, the small molecules described here inhibit both early and late events in the HIV replication cycle. In addition, PF-3450074 did not inhibit Gag particle production from HIV-1 transfected cells, suggesting that the compound series does not affect immature particle assembly. This is in contrast to the effects on immature particle assembly reported for NYAD-1. A co-crystal structure of a representative compound (PF-3450074) demonstrated a new binding site on HIV-1 CA distinct from those described for CAP-1 or CAI. Furthermore, PF-3450074 increased the rate of HIV-1 CA multimerization *in vitro*, while CAI and CAP-1 decreased the rate of CA multimerization in the same assay. While this does not necessarily define the action of these compounds on replicating virus, it does suggest a fundamentally different mechanism of inhibition from that of previously described CA inhibitors.

**Figure 6:**
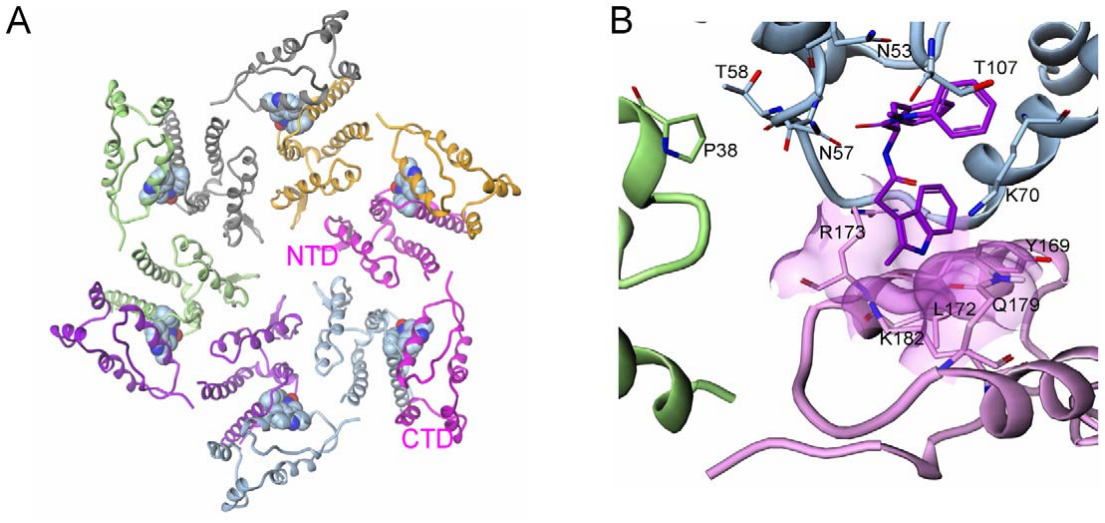
Model of inhibitor effects at the NTD-CTD interface. a) Model of HIV-1 capsid in hexameric complex with PF-3450074 bound in each of the binding pockets (each of the six full-length CA monomers are coloured differently); b) Close up view of model of interface between adjacent N-terminal subunits (green and blue) and the C-terminal domain of an adjacent monomer (pink) in assembled capsid. PF-3450074 highlighted in purple. Residues highlighted Pro38 (adjacent N-terminal domain green), Thr-107, Thr-58, Asn-57, Asn-53, Lys-70 (N-terminal domain blue), Tyr-169, Leu-172, Arg-173, Gln-179, Lys-182 (C-terminal domain pink).

The proposed mechanism of action for both the early and late stage activities of this new class of inhibitors involves a direct effect on higher-order structures of HIV CA, in assembly and uncoating. Although the present data do not indicate whether the compounds enhance or inhibit the uncoating process, either effect is likely to interfere with proper reverse transcription [25]. HIV-1 capsid mutations proximal to the PF-3450074 binding pocket have been described that either destabilize or enhance the stability of viral cores and result in specific postentry defects in virus replication [24]. It is possible that such mutations and the compounds described in this study have analogous effects on inter-subunit capsid interactions. To gain further insights into the mode of action of PF-3450074, we generated a model of an assembled capsid hexamer in complex with PF-3450074 (Fig. 6a) based on superpositioning of published assembled capsid structures [13], [14] with the structure of the PF-3450074/CA complex. In the model, the R3 indole group which protrudes from the NTD in our structure localizes to the interface between capsid monomers in an assembled capsid and sits directly between the NTD of one capsid monomer and the C-terminal domain of another, making contacts to Tyr-169, Leu-172, Arg-173, Gln-179, and Lys-182 (Fig. 6b). This suggests the R3 indole group of PF-3450074 could play a critical role in modulating inter-subunit interactions. Both the CA NTD contact residues described by the co-crystal structure and these putative C-terminal contacts are well conserved across viral strains (Tables S5 and S6). This is consistent with the broad-spectrum antiviral activity observed for this series.

Although the sum of our results suggests a mechanism that affects interactions between capsid monomers, the early stage activity is consistent with other models. Cyclophilin A plays a critical role in the early stages of HIV-1 replication through interactions with the viral capsid [26]. Also, capsid-binding restriction factors such as the tripartite motif containing (TRIM) proteins prevent the infection of many primate cells with HIV or SIVs from other species [5], [9]. Thus, based on our data, we cannot dismiss the possibility that, during early infection, this new series might affect specific capsid-host protein interactions that mediate the viral uncoating process. A detailed study of the early stage activity of PF-3450074 has demonstrated direct destabilization of the HIV-1 capsid and a dependence on cyclophilin A, indicating that the compound induces premature uncoating of the virus, potentially through a mechanism similar to that of TRIM restriction [27].

In this study, we identify a new binding site on HIV-1 CA that can be targeted by small molecule inhibitors resulting in broad-spectrum antiviral activity. In addition, we describe the discovery and characterization of a novel series of compounds that act at this site and inhibit the virus at two points in the replication cycle. This series should serve as a good starting point for the development of a new class of HIV therapeutics through structure-based drug design or other approaches. The broad spectrum activity of this series is particularly exciting and highlights this novel mechanism as a significant therapeutic opportunity.

## Material and Methods

### Cells, Virus, and Compounds

HeLa CD4 LTR/beta-Gal, MT-2, PM1, CEM-SS and HEK 293 cells as well as pNL4-3 HIV-1 infectious clone, HIV-1 IIIB, HIV-1 RF, HIV-1 BaL, and all primary isolates were obtained through the National Institutes of Health (NIH) AIDS Research and Reference Reagent Program, Bethesda, MD. JC53BL cells were sourced from Tranzyme. Efavirenz (EFV) was kindly provided by DuPont Merck (Wilmington, DE). Nelfinavir (NFV), 135137, PF-3450074, and PF-3759857 were synthesized by Pfizer Inc.

### Cytopathic Effect Assays (CPE)

As described [18], host cells were infected with HIV-1 NL4-3, HIV-1 IIIB, HIV-1 RF. The cytopathic effect was measured using XTT reagent and the therapeutic index (TI) calculated by dividing the CC_50_ (mock infected cells) value by the EC_50_.

### Clinical Isolate Antiviral Assays

PHA-stimulated PBMC's were incubated for 1 hour with virus at an moi of 0.001–0.01. They were plated in 96-well plates at 5×10^5^ cells/ml and incubated for 5 days at 37°C.10 µL of the supernatant was then transferred to a plate containing 40 µL of JC53BL cells at 0.5×10^5^ cells/ml. After 2 days, the cells were processed for β-galactosidase activity using the FluorAce kit (Bio-Rad). PF-3794231 was tested against clinical isolates at the Southern Research Institute (Frederick, MD) as previously described [18].

### Single-Cycle Infection Assays

The single-cycle infectious HIV-1 reporter viruses were generated as previously described [28] by co-transfecting HEK293 cells with an HIV-1 NL4-3 single-cycle infectious cDNA (pNL4-3deltaEnv) and an NL4-3 or VSV-G envelope expression vector. Half-log dilutions of test compounds were added to HeLa CD4 LTR/beta-Gal target cells, seeded in 96-well plates at a cell density of 1×10^4^ cells per well in DMEM containing 10% FBS. Compound-treated or compound-free target cells were then infected with the HIV-1 single-cycle infectious virus and after 72 hours measured for the induction of beta-galactosidase Data were expressed as the percent of reporter gene activity in infected compound-treated cells relative to that of infected, compound-free cells.

### Virus Production-Infectivity Assays

Envelope-deleted NL4-3 cDNA (pNL4-3deltaEnv) was co-transfected into HEK 293 cells with an HIV envelope expression vector as previously described [29]. Half-log dilutions of test compounds were then added to transfected cell cultures 3 hrs after transfection. The supernatants were harvested 72 hrs after transfection and infectious virus production was subsequently quantified by measuring the induction of the beta-galactosidase reporter gene after 100-fold dilution into fresh medium and incubation in the presence of HeLa CD4 LTR/beta-Gal target cells for 72 hours.

### Selection and Characterization of Resistant Virus

Viral variants resistant to PF-1385801 or PF-3759857 were selected as described previously [18]. To construct NL4-3 recombinant virus containing the Q67H, K70R, Q87P, T107N and L111I amino acid substitutions identified in the serial passage studies, viral cDNAs that had been TOPO cloned for sequence analysis were digested with BssHII and ApaI and ligated back into a wild type pNL4-3 background. The effect of amino acid substitutions in HIV-1 Gag sequences on PF-3759857 susceptibility was measured in HIV Replication assays as described previously [15].

### Protein Expression and Purification

Proteins were expressed in *E. Coli* BL21(DE3) in 2YT containing kanamycin. Cells were harvested following induction with IPTG and growth overnight at 20°C. CA_1-231_ was purified as described [30]. For CA_1-146/Δ87-99G_, cells were resuspended and lysed in buffer A (50 mM TrisHCl pH 7.4, 150 mM NaCl, 10 mM imidazole, and 2 mM β-mercaptoethanol). The lysate was clarified by centrifugation, filtered then applied to a His trap nickel affinity column (Sigma), eluting with buffer A supplemented with 300 mM imidazole. The eluted material was further purified on a Sephacryl 100HR column in 50 mM TrisHCl pH 7.4, 150 mM NaCl and 2 mM β-mercaptoethanol.

### HIV Capsid Multimerisation Assay

Multimerisation assays were performed as previously described [10]. Compound was added to 30 µg of full-length CA protein in 50 mM sodium phosphate buffer, pH 8.0 in a volume of 30 µl. Capsid assembly was initiated by addition of a concentrated NaCl solution (50 µl 5 M NaCl in 50 mM sodium phosphate, pH 8.0). Optical density was monitored on a Molecular Devices SpectraMax spectrophotometer at 350 nm every 20 s for 1 h.

### Transmission Electron Microscopy (TEM)

PBMCs were incubated with 7 µM PF-3450074 (approx. 10× IC_50_), for 30 minutes before being infected with mock virus (medium alone), or with NL4-3 at an MOI of 1. At 72 hours post-infection the supernatants were removed and the cells were fixed for 60 min in MacDowell's fixative (4% (v/v) paraformaldehyde, 1% glutaraldehyde in 0.1M phosphate Milliong buffer, pH 7.3) at 4 C. The cells were rinsed in 0.15M Phosphate Sörensen buffer with 0.2% (v/v) NaCl (pH 7.3) and suspended in warm 3.5% agar in water. The agar blocks were cooled to 4°C and washed three times for 10 minutes in 0.15M Phosphate Sörensen buffer with 0.2% (v/v) NaCl (pH 7.3). The agar blocks with the cells were stained using 2% Osmium tetroxide in 0.3M phosphate Sörensen buffer (pH 7.4) for 1 h at 4°C. The blocks were washed 4 times for 10 minutes in sterilized water at room temperature before they were dehydrated in graded ethanol (50%, 70%, 90%, 100%, 100%) for 15 minutes at each concentration at room temperature. The blocks were treated with propylene oxide three times for 15 minutes in room temperature before the resin was infiltrated for 1 hour at room temperature with a 1∶1 propylene oxide∶Epon mix (Epon contains epoxy embedding medium 20 mL (Epon 812 resin)) and 12 ml MNA (methyl nadic anhydride) and 9 ml DDSA (dodecenyl succinic anhydride)) before the resin was treated in epon overnight at room temperature. The resin was then embedded with Epon and BDMA 3% for 1 hour at room temperature. The resin was polymerised for 48h at 58–60°C. Approximately 1 micron thick semi-thin sections were cut and stained in toluidine blue and observed in light microscopy. Subsequently, ultra-thin sections of 70–90 nm were cut on an Ultracut E Reichert microtone, and stained with uranyl acetate and lead citrate. Observations were made using a Jeol 1200 EX II electron microscope.

### Isothermal Calorimetry

Isothermal titration calorimetry (ITC) was performed using the VP-ITC calorimetric system (GE Healthcare). Protein solutions for ITC were dialyzed against buffer A (50 mM TrisHCl pH 7.5/150 mM NaCl/2 mM β-mercaptoethanol). The dialyzed protein solution (15 µM) in the calorimetric cell (1.4274 ml) was titrated at 25°C with ligand (200 µM) in the buffer A using 1×2 µL, followed by 25×10 µL injections. Heat evolved was obtained from the integral of the calorimetric signal and heat of dilution was negligible in titrations of the ligand into buffer only. Analysis was carried out with Origin 5.0 software (GE Healthcare). Binding parameters such as the number of binding sites (*n*), the binding constant (*K*_a_, M^−1^), and the binding enthalpy (Δ*H*_a_, kcal/mol of bound ligand) were determined by fitting the experimental binding isotherms.

### Protein Crystallization, Data Collection, and Structure Refinement

Protein was concentrated to 30 mg/ml and inhibitor added to 5 mM from a 100 mM DMSO stock solution. After standing for 2 hours, hanging drop crystallizations were set up consisting of 2 µl of protein and 1 µl of well solution (20% PEG 8000, 100 mM phosphate-citrate pH 4.2 and 200 mM sodium chloride). Crystals grew overnight at room temperature and were frozen for X-ray data collection following addition of 60% NDSB containing 40% ethylene glycol (4 µl). Data was collected at the ESRF (ID14-4) on an ADSC Q315 detector, integrated with mosflm [31] and scaled with CCP4 package SCALA [32]. The structure was solved by molecular replacement using MOLREP [32] with a truncated model of the N-terminal CA. The structure was refitted using QUANTA version 2000.1 (Accelrys Inc., San Diego, CA) and refinement was carried out using REFMAC [33]. Data collection and refinement statistics are shown in Table S7. Coordinates are stored in the Protein Data Bank (PDB ID code 2XDE).

## Supporting Information

Table S1 In Vitro Antiviral Activity of PF-3450074 Against Different HIV-1 Clinical Isolates or Laboratory Strains in PBMCs
(0.08 MB PDF)

Table S2 In Vitro Antiviral Activity of PF-3759857 Against Different HIV-1 Clinical Isolates or Laboratory Strains in PBMCs
(0.09 MB PDF)

Table S3 In Vitro Antiviral Activity of Efavirenz Against Different HIV-1 Clinical Isolates or Laboratory Strains in PBMCs
(0.08 MB PDF)

Table S4 In Vitro Antiviral Activity of AZT Against Different HIV-1 Clinical Isolates or Laboratory Strains in PBMCs
(0.09 MB PDF)

Table S5 Conservation of capsid binding site residues across HIV-1 strains
(0.08 MB PDF)

Table S6 Conservation of capsid binding site residues across HIV-2 strains
(0.08 MB PDF)

Table S7 Crystallographic Data Collection and Refinement Statistics
(0.06 MB PDF)

Figure S1 **Effect of PF-3450071 on proteolytic processing of HIV-1 Gag.** For the Western blot analyses, HEK 293 cells were transfected with pNL4-3 in the presence or absence of compound, and supernatants were harvested 72h later. Infectious virus production was measured using a portion of the supernatants of transfected cells in virus production/infection assays as described in materials and methods. Western blot of the supernatants was generated as previously described in reference 17. Virus expression in the presence of the protease inhibitor NFV displays an array of unprocessed forms of the Gag polyprotein, however PF-3450071, has no effect on proteolytic processing of Gag, even at highly inhibitory concentrations.
(0.05 MB PDF)

Figure S2 Structure of PF-4159193
(0.00 MB PDF)
