# Supplementary material for: HIV Capsid is a Tractable Target for Small Molecule Therapeutic Intervention
Source: PLoS Pathog. 2010 Dec 9;6(12):e1001220. doi: 10.1371/journal.ppat.1001220 (PMC3000358; doi:10.1371/journal.ppat.1001220)
Supplement: Table S1 — In Vitro Antiviral Activity of PF-3450074 Against Different HIV-1 Clinical Isolates or Laboratory Strains in PBMCs (0.08 MB PDF) [file ppat.1001220.s001.pdf]

Table S1: *In Vitro* Antiviral Activity of PF-3450074 Against Different HIV-1 Clinical Isolates or Laboratory Strains in PBMCs[a]. Supplementary to Figure 1, the properties of the various isolates tested (represented by single points) against the compound are shown.

HIV-1 Isolate	Clade[b]	Receptor	EC_{50} (μM)	TI[c]
NL4-3	B	X4	0.64 ± 0.10	> 16
BaL	B	R5	0.26 ± 0.08	> 38
SF162	B	R5	0.25 ± 0.11	> 40
92HT599	B	R5/X4	0.08 (0.04, 0.12)	> 25
92HT596	B	R5/X4	0.25 (0.38, 0.12)	> 26
92BR004	B	R5	0.19 (0.34, 0.04)	> 29
92UG037	A	R5	0.32 ± 0.18	> 32
94UG118	D	R5	0.23 (0.14, 0.33)	> 31
97ZA003	C	R5	0.20 (0.19, 0.21)	> 47
CMU06	E	X4	0.14 (0.16, 0.12)	> 61

[a]Antiviral activity was determined by measuring RT activity 7 days after infection of PBMCs with HIV-1 clinical isolates (X4, CXCR4; R5, CCR5).

[b]Clade and receptor assignments based on designations from the NIH AIDS Research and Reference Reagent Program or our collaborator for the study (SRI).

[c]TI, therapeutic index calculated by dividing the mean EC_{50} value into the mean CC_{50} value determined in PBMCs (>10 microM).
