# Supplementary material for: HIV Capsid is a Tractable Target for Small Molecule Therapeutic Intervention
Source: PLoS Pathog. 2010 Dec 9;6(12):e1001220. doi: 10.1371/journal.ppat.1001220 (PMC3000358; doi:10.1371/journal.ppat.1001220)
Supplement: Table S2 — In Vitro Antiviral Activity of PF-3759857 Against Different HIV-1 Clinical Isolates or Laboratory Strains in PBMCs (0.09 MB PDF) [file ppat.1001220.s002.pdf]

Table S2: *In Vitro* Antiviral Activity of PF-3759857 Against Different HIV-1 Clinical Isolates or Laboratory Strains in PBMCs[a]. Supplementary to Figure 1, the properties of the various isolates tested (represented by single points) against the compound are shown.

**HIV-1 Isolate**	**Clade[b]**	**Receptor**	**EC_{50} (μM)**	**TI[c]**
IIIB	B	X4	1.36	>73.7
JR-CSF	B	R5	1.19	>84.0
92BR014	B	R5/X4	3.17	>31.5
92BR021	B	R5	1.25	>80.0
92BR025	C	R5	1.08	>92.4
92BR020	B	R5	1.55	>64.7
92RW016	A	R5	1.14	>87.9
92TH014	B	R5	1.34	>74.6
92TH026	B	R5	1.94	>51.4
93BR017	B	R5	1.62	>61.9
93BR020	F	R5/X4	1.03	>97.1
93BR021	B	R5	0.60	>166
93BR023	B	R5	0.77	>130
96USHIPS7	B	R5	1.03	>97.1
91US056	B	R5	1.76	>56.8
92US076	B	R5	1.03	>96.8
98IN017	C	X4	0.51	>196
93IN101	C	R5	1.01	>99.3

[a]Antiviral activity was determined by measuring RT activity 7 days after infection of PBMCs with HIV-1 clinical isolates. (X4, CXCR4; R5, CCR5).

[b]Clade and receptor assignments based on designations from the NIH AIDS Research and Reference Reagent Program or our collaborator for the study (SRI).

[c]TI, therapeutic index calculated by dividing the mean EC_{50} value into the mean CC_{50} value determined in PBMCs (>100 microM).
