# Supplementary material for: HIV Capsid is a Tractable Target for Small Molecule Therapeutic Intervention
Source: PLoS Pathog. 2010 Dec 9;6(12):e1001220. doi: 10.1371/journal.ppat.1001220 (PMC3000358; doi:10.1371/journal.ppat.1001220)
Supplement: Table S3 — In Vitro Antiviral Activity of Efavirenz Against Different HIV-1 Clinical Isolates or Laboratory Strains in PBMCs (0.08 MB PDF) [file ppat.1001220.s003.pdf]

Table S3: *In Vitro* Antiviral Activity of Efavirenz Against Different HIV-1 Clinical Isolates or Laboratory Strains in PBMCs[a]**.** Supplementary to Figure 1, the properties of the various isolates tested (represented by single points) against the compound are shown.

HIV-1 Isolate	Clade[b]	Receptor	EC_{50} (nM)
NL4-3	B	X4	1.2 ± 1.64
BaL	B	R5	1.54 ± 2.41
SF162	B	R5	0.76 ± 0.8
92HT599	B	R5/X4	0.79 (0.79, 0.8)
92HT596	B	R5/X4	0.11 (0.2, 0.058)
92BR004	B	R5	0.096 ± 0.096
92UG037	A	R5	0.27 ± 0.299
94UG118	D	R5	0.22 ±0.064
97ZA003	C	R5	0.021 (0.041, 0.011)
CMU06	E	X4	0.35 ± 0.12
