# Supplementary material for: HIV Capsid is a Tractable Target for Small Molecule Therapeutic Intervention
Source: PLoS Pathog. 2010 Dec 9;6(12):e1001220. doi: 10.1371/journal.ppat.1001220 (PMC3000358; doi:10.1371/journal.ppat.1001220)
Supplement: Table S4 — In Vitro Antiviral Activity of AZT Against Different HIV-1 Clinical Isolates or Laboratory Strains in PBMCs (0.09 MB PDF) [file ppat.1001220.s004.pdf]

Table S4: *In Vitro* Antiviral Activity of AZT Against Different HIV-1 Clinical Isolates or Laboratory Strains in PBMCs**.** Supplementary to Figure 1, the properties of the various isolates tested (represented by single points) against the compound are shown.

HIV-1 Isolate	Clade	Receptor	EC_{50} (nM)	TI
IIIB	B	X4	13.2	>76
JR-CSF	B	R5	5.49	>182
92BR014	B	R5/X4	3.02	>331
92BR021	B	R5	20.7	>48
92BR025	C	R5	9.92	>101
92BR020	B	R5	9.58	>104
92RW016	A	R5	17.2	>58
92TH014	B	R5	7.65	>131
92TH026	B	R5	64.6	>15
93BR017	B	R5	15.5	>65
93BR020	F	R5/X4	489	>2
93BR021	B	R5	5.44	>184
93BR023	B	R5	3.46	>289
96USHIPS7	B	R5	7.75	>129
91US056	B	R5	18.1	>55
92US076	B	R5	9.37	>107
98IN017	C	X4	51.0	>20
93IN101	C	R5	17.9	>56
