# Supplementary material for: HIV Capsid is a Tractable Target for Small Molecule Therapeutic Intervention
Source: PLoS Pathog. 2010 Dec 9;6(12):e1001220. doi: 10.1371/journal.ppat.1001220 (PMC3000358; doi:10.1371/journal.ppat.1001220)
Supplement: Table S5 — Conservation of capsid binding site residues across HIV-1 strains (0.08 MB PDF) [file ppat.1001220.s005.pdf]

**Table S5:** Conservation of capsid binding site residues across HIV-1 strains

| *N-terminal domain binding site residues* | | |
|---|---|---|
| **Residue** | **Conservation** | **Other residues (% observed)** |
| N53 | 99.4% | X(0.6%) |
| L56 | 99.4% | X(0.6%) |
| N57 | 99.4% | X(0.6%) |
| V59 | 99.4% | I(0.6%) |
| Q63 | 100% | - |
| M66 | 95.2% | L(4.8%)* |
| Q67 | 98.8% | E(1.2%) |
| L69 | 100% | - |
| K70 | 99.4% | E(0.6%)* |
| I73 | 100% | - |
| A105 | 98.8% | X(0.6%), V(0.6%) |
| T107 | 96.4% | A(1.8%), S(1.8%) |
| Y130 | 100% | - |
| | | |
| *Putative C-terminal contact residues* | | |
| **Residue** | **Conservation** | **Other residues (% observed)** |
| Y169 | 35% | F(65%) |
| L172 | 99.4% | I(0.6%) |
| R173 | 100% | - |
| Q179 | 99.4% | H(0.6%) |
| K182 | 100% | - |

Sequence data taken from 2009 HIV-1 sequence compendium (http://www.hiv.lanl.gov/content/sequence/HIV/COMPENDIUM/2009compendium.html) comprising 164 representative sequences from different viral strains

*All from O genotype
