# Supplementary material for: HIV Capsid is a Tractable Target for Small Molecule Therapeutic Intervention
Source: PLoS Pathog. 2010 Dec 9;6(12):e1001220. doi: 10.1371/journal.ppat.1001220 (PMC3000358; doi:10.1371/journal.ppat.1001220)
Supplement: Table S6 — Conservation of capsid binding site residues across HIV-2 strains (0.08 MB PDF) [file ppat.1001220.s006.pdf]

Table S6: Conservation of capsid binding site residues across HIV-2 strains

N-terminal domain binding site residues		
**Residue in HIV-1**	**Conservation**	**Other residues (% observed)**
N53	100%	-
L56	100%	-
N57	100%	-
V59	100%	-
Q63	100%	-
M66	100%	-
Q67	100%	-
L69	100%	-
K70	0%	R(100%)
I73	95.7%	V(4.3%)
A105	100%	-
T107	100%	-
Y130	100%	-
Putative C-terminal contact residues		
**Residue**	**Conservation**	**Other residues (% observed)**
Y169	100%	
L172	95.7%	X(4.3%)
R173	100%	-
Q179	0	P(87%), A(8.7%), T(4.3%)
K182	95.7%	R(4.3%)

Sequence data taken from 2009 HIV-1 sequence compendium: (http://www.hiv.lanl.gov/content/sequence/HIV/COMPENDIUM/2009compendium.html) comprising 23 representative sequences from different viral strains.
