# Supplementary material for: HIV Capsid is a Tractable Target for Small Molecule Therapeutic Intervention
Source: PLoS Pathog. 2010 Dec 9;6(12):e1001220. doi: 10.1371/journal.ppat.1001220 (PMC3000358; doi:10.1371/journal.ppat.1001220)
Supplement: Table S7 — Crystallographic Data Collection and Refinement Statistics (0.06 MB PDF) [file ppat.1001220.s007.pdf]

Table S7: Crystallographic Data Collection and Refinement Statistics

	**PF-3450074**
ESRF Beamline	ID14-4
Temperature (K)	100
Wavelength (Å)	0.979
Space Group	$P2_1$
Cell constants (Å)	a=39.7559
	b=71.0349
	c=41.4188
	β=90.4092
Resolution (Å)*	1.4 (1.48-1.4)
Completeness (%)	98.8 (99.4)
Rsym (%)	5.5 (6.4)
Multiplicity	3.4 (3.4)
I/σ(I)	14.1 (1.7)
Total reflections	152751 (22508)
Unique reflections	44735 (6570)
Resolution range (Å)	29.7 – 1.4
Amino acid residues	323
Water molecules	516
Ligand molecules	1
rmsd bond lengths (Å)	0.010
rmsd bond angles (°)	1.474
R factor (%)	19.5
Free R-factor (5%)	25.4

* Number in parentheses is the statistic for the highest resolution shell
