# Supplementary figures and images for: HIV Capsid is a Tractable Target for Small Molecule Therapeutic Intervention
Source: PLoS Pathog. 2010 Dec 9;6(12):e1001220. doi: 10.1371/journal.ppat.1001220 (PMC3000358; doi:10.1371/journal.ppat.1001220)

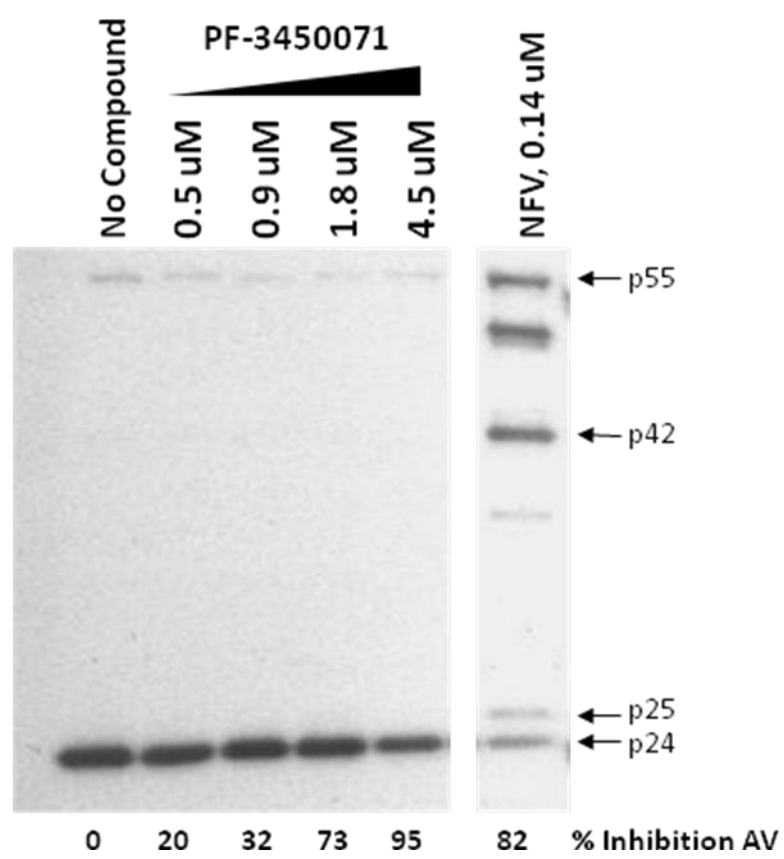

Supplement: Figure S1 — Effect of PF-3450071 on proteolytic processing of HIV-1 Gag. For the Western blot analyses, HEK 293 cells were transfected with pNL4-3 in the presence or absence of compound, and supernatants were harvested 72h later. Infectious virus production was measured using a portion of the supernatants of transfected cells in virus production/infection assays as described in materials and methods. Western blot of the supernatants was generated as previously described in reference 17. Virus expression in the presence of the protease inhibitor NFV displays an array of unprocessed forms of the Gag polyprotein, however PF-3450071, has no effect on proteolytic processing of Gag, even at highly inhibitory concentrations. (0.05 MB PDF) [file ppat.1001220.s008.pdf]

**Fig. S2:** Structure of PF-4159193

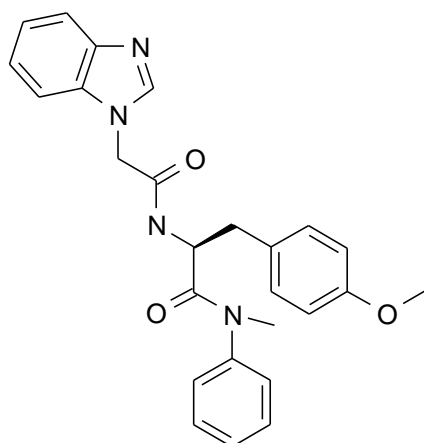

Supplement: Figure S2 — Structure of PF-4159193 (0.00 MB PDF) [file ppat.1001220.s009.pdf]
